# Hypothalamic control of puberty: from neuronal circuits to mechanisms for its metabolic regulation

**DOI:** 10.1007/s11154-025-10001-w

**Published:** 2025-10-18

**Authors:** Manuel Jimenez-Puyer, Verónica Sobrino, William H. Colledge, Susan Jones, Manuel Tena-Sempere

**Affiliations:** 1 Department of Cell Biology, Physiology and Immunology, University of Córdoba, Córdoba, 14004 Spain; 2 Instituto Maimónides de Investigación Biomédica de Córdoba (IMIBIC), Córdoba, 14004 Spain; 3 Department of Physiology, Development and Neuroscience, University of Cambridge, Cambridge, UK; 4 Hospital Universitario Reina Sofia, Córdoba, 14004 Spain; 5 CIBER Fisiopatología de La Obesidad y Nutrición, Instituto de Salud Carlos III, Córdoba, 14004 Spain

**Keywords:** Puberty, Reproduction, Hypothalamus, GnRH, Kiss1, Kisspeptins, Leptin, Metabolism

## Abstract

The hypothalamus is a singular brain region with essential roles in the control of a wide diversity of vegetative functions, from growth and energy balance to reproduction. These processes are governed by interconnected neuroendocrine pathways that enable proper adjustment of fundamental biological programs to internal and external cues along the lifespan. Puberty is a key maturational phenomenon that permits full sexual and somatic maturation, and attainment of reproductive capacity, together with important psychological changes. Puberty is to a large extent, a brain-driven phenomenon, with the hypothalamus playing a major role as the essential hub for the integration of central and peripheral signals, responsible for driving puberty onset and its modulation by endogenous and environmental factors. Our understanding of the hypothalamic circuits governing puberty has expanded enormously in the last decades, as exemplified by the discovery and later characterization of the roles of neurons producing kisspeptins, aka Kiss1 neurons, as major gatekeepers of puberty onset, mainly through their role as indispensable upstream activators of GnRH neurons. In recent years, the intimate molecular programs and co-players of Kiss1 neurons that participate in pubertal control have been partially exposed. In addition, given the paramount importance of metabolic signals in the modulation of puberty, the nature and mechanisms of action of different factors, converging at the hypothalamus, that participate in pubertal modulation by the metabolic and nutritional status have been disclosed. While characterization of these regulatory circuits is still uncomplete, this review aims to provide a synoptic and updated view of our current knowledge of the essential elements responsible for the hypothalamic control of puberty, also as a means to understand the putative basis for acquired pubertal disorders, including those linked to metabolic perturbations, such as early-onset obesity or undernutrition.

## Introduction: puberty as brain-controlled key neuroendocrine event

In mammals, timed somatic and reproductive maturation is a key developmental phenomenon. While essential maturational events take place in utero, a substantial component of the developmental program that leads to the attainment of reproductive capacity occurs postnatally and is culminated at puberty, the critical period when sexual maturity is achieved [1, 2]. Of note, puberty is a maturational continuum that not only entails the acquisition of fertility, but involves also important somatic changes, including completion of linear growth, as well as substantial psychological modifications, all leading to the adult phenotype [2, 3]. As an intricate, multi-factorial process, puberty is subjected to sophisticated regulatory programs, that integrate genetic determinants with endogenous and environmental cues, to ensure proper pubertal timing and completion [2]. While multiple signals participate in this phenomenon, compelling evidence points out that puberty is ultimately governed by brain-based mechanisms, that converge at the hypothalamus, as key brain center for vegetative control [4]. Indeed, considering its fundamental role in the integral control of multiple bodily functions [5, 6], from growth and metabolic homeostasis to reproduction, that are at the core of pubertal maturation, the hypothalamus is ideally suited to operate as major integratory hub, holding the essential circuits for the precise control of puberty.

In terms of reproductive maturation, which will be the major focus of this review, puberty is characterized by the complete functional activation of the so-called hypothalamic-pituitary–gonadal (HPG) axis. This complex neuroendocrine system is organized into three major levels that are interconnected, via feedforward and feedback loops, by the following signals: the decapeptide, gonadotropin-releasing hormone (GnRH) at the hypothalamus; the pituitary gonadotropins, luteinizing hormone (LH) and follicle-stimulating hormone (FSH); and the gonadal hormones, of steroid and peptidergic nature [4]. Importantly, the major hierarchical element of this system is GnRH, produced by a scarce population of neurons scatteredly located mainly at the rostral hypothalamus [4]. These operate as the final output signal for the brain control of the reproductive axis and its full activation at puberty, thus illustrating the fundamental role of hypothalamic-born signals in pubertal maturation. Indeed, enhancement of the neurosecretory activity of GnRH neurons dictates the prepubertal rise of the pulsatile secretion of gonadotropins, driving the increase of the gonadotropic input to the gonads [4]. In turn, gonadal hormones feedback mainly at the level of the hypothalamus, to restrain (i.e., negative feedback) the release of GnRH and gonadotropins into proper pulsatile patterns in both sexes, and to trigger (i.e., positive feedback) the preovulatory surge of gonadotropins, responsible for ovulation in females [7].

The biological and medical relevance of puberty is remarkable, not only as a life-changing event in the vital course of any individual, but also because of its sensitivity to multiple external and internal cues and its potential influence on the health status or disease risk at later stages of life. This has propelled very active research on the pathophysiology of puberty, including experimental, genetic and clinical studies, aiming to unveil the neuroendocrine mechanisms that control puberty and their eventual perturbations. Given the breadth of potential content, in this scoping review, we will concentrate on recent developments in our knowledge of the hypothalamic circuits that govern puberty, with a major focus on key neuropeptide pathways as well as mechanisms for its metabolic control and alterations in adverse nutritional conditions.

## Hypothalamic circuits and the control of puberty – pivotal roles of GnRH and Kiss1 neurons

Among the hypothalamic circuits regulating puberty, key neuronal components can be singled out given their essential roles in pubertal maturation, as major regulatory elements and indispensable output signals for the brain control of puberty. Due to their paramount relevance, the roles of GnRH and Kiss1 neurons in pubertal maturation will be reviewed in this section.

### Key role of GnRH neurons in pubertal maturation

**Fig. 1:**
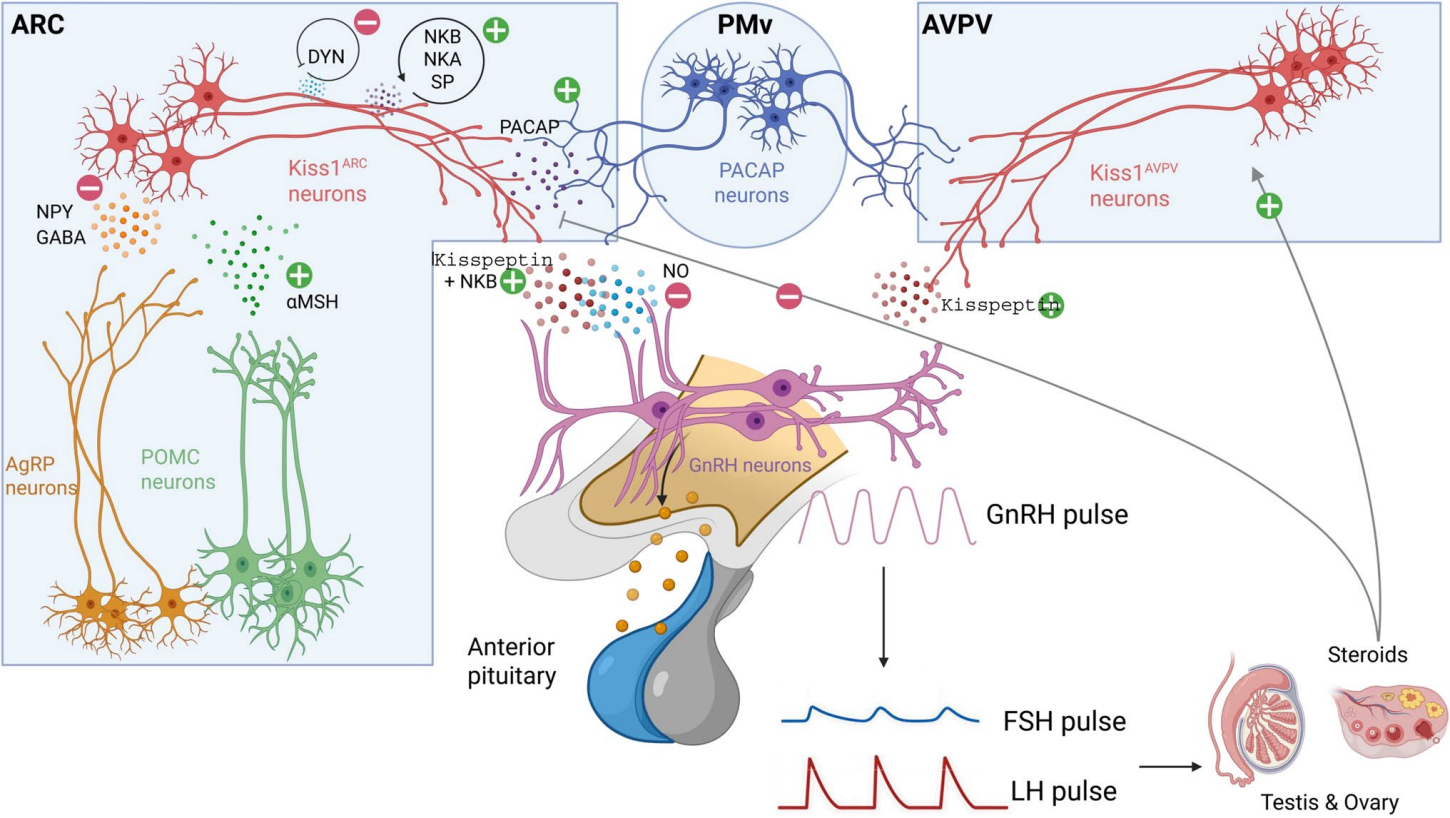
Overview of hypothalamic networks controlling puberty. Schematic representation of the main neuroendocrine elements involved in the control of puberty. GnRH neurons, as major output pathway for the control of the reproductive axis, are responsible for the pulsatile release of GnRH, which dictates the secretory patterns of pituitary gonadotropins, LH and FSH. In turn, GnRH neurosecretory activity is governed by a complex network of upstream excitatory and inhibitory input; in this network, Kiss1 neurons, located in the arcuate nucleus (ARC) and the anteroventral periventricular nucleus (AVPV), play very prominent roles by releasing kisspeptins, which are also the target of the feedback effects of gonadal steroids. Other neuropeptides, such as neurokinin B (NKB) and dynorphin (Dyn), as well as transmitters, such as nitric oxide (NO), participate also in these hypothalamic circuits. Neuronal afferents to Kiss1 neurons, with prominent roles in metabolic control, such as AgRP and POMC neurons in the ARC, and PACAP neurons from the PMv are also depicted. For further details, see Sects. 2 and 4. Created with BioRender®

Given their pivotal position in the hierarchy of the HPG axis, a fundamental question to understand the neuroendocrine basis of puberty is to define the mechanisms whereby GnRH neurons attain a maximal neurosecretory state during pubertal maturation. In fact, it was long debated whether puberty is triggered by changes in the sensitivity of GnRH neurons to the negative feedback actions of sex steroids (i.e., the gonadostat hypothesis), or it is driven by the dynamic fluctuation of central inputs that ultimately control GnRH neurosecretion [8]. Demonstration of the lack of substantial changes in the sensitivity to negative feedback prior to puberty [8], together with the solid evidence garnered in recent years of important changes in key afferent regulators of GnRH neurons during the pubertal transition [2], have set the contention that puberty onset is primarily driven by the concerted operation of key central networks controlling GnRH neurons [3]. These changes actually involve multiple transmitters and molecular mediators, either excitatory or inhibitory, which originate and/or act at both neuronal and non-neuronal (e.g., glial) afferents (Fig. 1). In this scenario, the current consensus is that, rather than the result of a unique trigger, puberty is set in motion by the sequential activation or inactivation of cascades of excitatory and inhibitory pathways that ultimately converge and modulate the activity of GnRH neurons [9, 10]. This core regulatory function is conducted within the hypothalamus, but is influenced by extra-hypothalamic and peripheral-born signals, as well as other regulatory factors, including key elements of the neurovascular unit modulating median eminence permeability [11].

From a neuroendocrine perspective, pubertal maturation relies in the timed re-awakening of the pulsatile secretion of gonadotropins, after a quiescent infantile period, which is particularly evident in primates. This patterned secretory profile is dictated by the pulsatile release of GnRH that, despite some intrinsic oscillatory activity of GnRH neurons themselves, is mainly driven by a diversity of upstream afferents, of excitatory or inhibitory nature, that integrate at the so-called GnRH pulse generator. Within the multi-layered regulatory circuits that form the pulse generator, compelling evidence, including solid neuroanatomical and physiological data, has pointed out that Kiss1 neurons located in the hypothalamic arcuate nucleus (ARC) play a dominant role [12, 13] as the principal stimulatory afferent to GnRH neurons. Considering the paramount importance of Kiss1 neurons and kisspeptins in the control of puberty and other essential aspects of reproductive maturation and function, the major features of this neuronal system will be reviewed in the following subsection (see also Fig. 1).

### Essential roles of Kiss1 neurons in the control of puberty

Kisspeptins are a family of neuropeptides encoded by the *Kiss1* gene that operate via the G-protein couple receptor, Gpr54. Initially catalogued as putative metastasis-suppressors, the fundamental roles of the so-called Kiss1 system in the control of puberty was first recognized in late 2003, when inactivating mutations of the receptor were found to cause a profound state of central hypogonadism and lack of pubertal maturation [14, 15]. Kisspeptins have been found to be produced primarily by two distinct populations of Kiss1 neurons located in the hypothalamus: one located in the ARC (Kiss1^ARC^ neurons), and the population placed in the rostral hypothalamic area, mainly in the anteroventral periventricular area (AVPV), as characterized in detail in rodents (Kiss1^AVPV^ neurons). While Kiss1^ARC^ neurons are highly conserved in both sexes in multiple species, including mammals [16, 17], the population of Kiss1^AVPV^ neurons is more prominently developed in females, and has been connected with the central mechanisms driving ovulation. In fact, ARC and AVPV Kiss1 neurons are differentially modulated by key reproductive signals (e.g., sex steroids) and have been proposed to play different roles in the control of key aspects of the reproductive axis, such as the negative (channeled via Kiss1^ARC^ neurons) and positive (via Kiss1^AVPV^ neurons) feedback control of gonadotropins [4, 18]. Notably, these populations produce different sets of co-transmitters, further illustrating their functional divergence. Thus, a majority of Kiss1^ARC^ neurons co-express neurokinin B (NKB; encoded by *Tac2* in rodents) and dynorphin (Dyn; encoded by *Pdyn*), and therefore have been named KNDy (for the expression of Kiss1, NKB and Dyn) [19]. In contrast, Kiss1^AVPV^ neurons do not produce NKB but in mice co-express tyrosine hydroxylase, key enzyme for catecholamine synthesis [20].

Dissection of the specific roles of these Kiss1 neuronal populations in the precise control of puberty remains incomplete, although it has been approached in recent years by a combination of expression and functional analyses. Neuroanatomical analyses documented that Kiss1^ARC^ neurons are present at birth but are not fully functional until puberty, while Kiss1^AVPV^ expression starts to increase around postnatal day (PND) 15 in rats and reaches adult levels around puberty [21]. While the maturational programs of Kiss1 neurons during puberty will be summarized in the following section, it is worth mentioning that virogenetic and functional genomics have been applied recently to tease apart the individual contribution of kisspeptins produced by Kiss1^ARC^ and Kiss1^AVPV^ neurons. Thus, using an adeno-associated virus (AAV)-driven antisense *Kiss1* vector, it was shown that partial suppression of *Kiss1* expression in rats deferred vaginal opening, an external sign of puberty onset, when targeting Kiss1^AVPV^, but not Kiss1^ARC^ neurons [22]. It must be noted, however, that this strategy induced only a modest decrease in kisspeptin content, as measured by immunoassays, that accounted only for a 32% reduction in the ARC and 37% in AVPV, suggesting that this degree of suppression might be insufficient to disclose the actual roles of the Kiss1^ARC^ population. More recently, strategies to ablate Kiss1 selectively from KNDy neurons (but not from non-KNDy Kiss1 cells) have been implemented in mice using Cre-loxP approaches in Kiss1^loxP/loxP^ mice crossed with either Tac2-Cre or Pdyn-Cre expressing lines. These studies revealed that kisspeptins produced by Tac2-expressing cells (i.e., co-expressing kisspeptins and NKB) are dispensable for puberty onset in both males and females, as putatively compensated by kisspeptins from other non-KNDy cells. Interestingly, the suppression of ARC *Kiss1* expression was much greater in females, which displayed signs of accelerated reproductive senescence and premature ovarian insufficiency after puberty, whereas males remained grossly unaffected, due to retention of nearly 50% *Kiss1* expression in the ARC [23]. In the same line, ablation strategies using Pdyn-Cre to target KNDy cells failed to demonstrate detectable alterations in the timing of puberty in male or female mice; yet, in this case, the post-pubertal impact of *Kiss1* ablation affected reproductive function in both sexes [24]. While these findings collectively point out a dispensable role of KNDy-born kisspeptins in pubertal maturation, the co-existence of Kiss1-only cells in the ARC, the persistent expression of other KNDy peptides (e.g., NKB) in the above models of conditional *Kiss1* KO, and the input from other Kiss1 neuronal populations outside the ARC can help to reconcile these findings with previous solid literature supporting that inactivation of Kiss1 signaling leads to pubertal disruption [18]. In good agreement, a recent study, using diphtheria toxin-mediated ablation of Kiss1 neurons at a temporal window (early postnatal) with predominance of Kiss1^ARC^ neurons [21], strongly suggested that this neuronal population is key for proper pubertal timing [25]. It must be noted, though, that in contrast to gene ablation, cell ablation strategies disrupt the whole repertoire of cellular signals, and this might explain the differences detected across the above mouse lines. To our knowledge, no genetic models for the specific targeting of Kiss1^AVPV^ neurons to address their pubertal (or reproductive) actions have been reported to date.

Besides their projections to GnRH neurons, Kiss1^ARC^ and Kiss1^AVPV^ neurons have been shown to project to other intra- and extra-hypothalamic areas, including putative interconnections between the ARC and AVPV, whose relevance in the specific control of puberty is yet to be fully clarified. In addition, other populations of Kiss1 neurons have been described, whose role in pubertal modulation remains ill defined. This is clearly exemplified by the population of Kiss1 neurons placed in the medial amygdala [26]. In the context of puberty, it has been documented that *Kiss1* expression in this region is negligible in juvenile male mice, but substantially increases during late puberty, coinciding with the rise of circulating sex steroid levels [27], thus suggesting that this phenomenon might not be a primary driver for puberty, but instead a consequence of the elevation of sex steroid levels at the initial pubertal stages. In any event, it has been shown that antagonism of kisspeptin signaling in the posterodorsal region of the medial amygdala during the juvenile period deferred the onset of puberty in female rats [28]. Of note, lesions of the medial amygdala are known to advance puberty onset [29]; yet, the eventual contribution of Kiss1 neurons to that phenomenon is yet to be defined.

## Maturational programs and key regulatory mechanisms of Kiss1 neurons during puberty

**Fig. 2:**
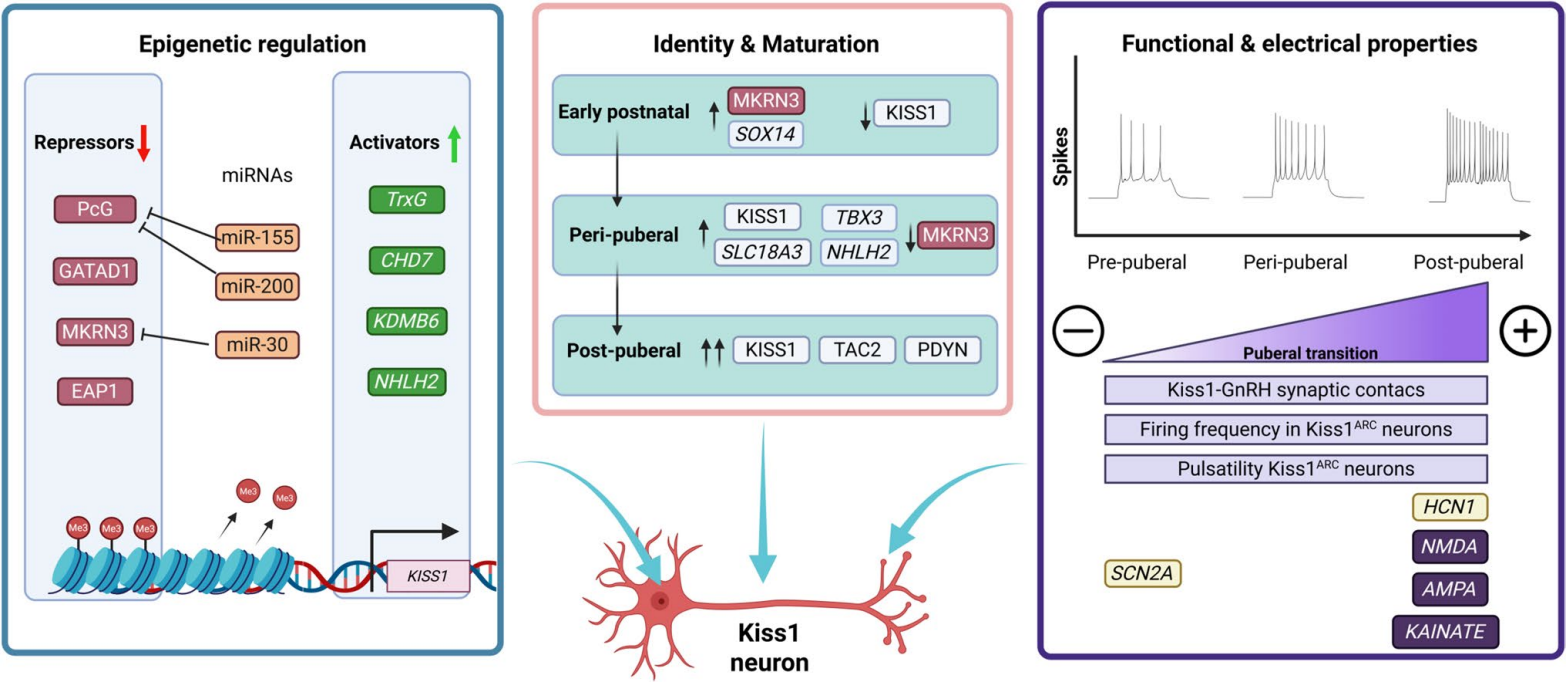
Developmental programs and regulatory mechanisms of Kiss1 neurons during puberty. Kiss1 neurons, as a pivotal component of the hypothalamic networks controlling puberty, are subjected to a complex maturational program, that involves different epigenetic regulatory mechanisms, together with the action of essential factors for attaining and maintaining cell identity and changes in their functional and electrical properties neurons during pubertal maturation. For further details, see Sect. 3. Created with BioRender®

Compelling evidence, gathered more than a decade ago, conclusively demonstrated that Kiss1 neurons undergo a complex maturational program during postnatal development and pubertal transition, which seems to be essential to secure an appropriate kisspeptin input to guarantee pubertal transition. This developmental process not only involves an increase in the populations of Kiss1 neurons (see Sect. 2), but also of the number of their projections and appositions onto GnRH neurons [30], where kisspeptins have been shown to elicit enhanced firing responses across pubertal maturation [31, 32]. These concerted maturational events lead to enhanced kisspeptin tone, as major driver of pubertal progression. In good agreement, intracerebroventricular injections of a kisspeptin antagonist has been shown to delay pubertal timing in female rats [33]. In recent years, our knowledge about the molecular underpinnings for some of the above maturational changes have been partially exposed. In addition, further characterization of the functional properties and electrical patterns of activity of Kiss1 neurons, also during puberty, have begun to be defined, as summarized in the following subsections (Fig. 2).

### Global transcriptional changes and key transcriptional factors in Kiss1 neurons at puberty

While initial studies focused on the analysis of changes of expression of specific transcripts, including *Kiss1* itself, in Kiss1 neurons during puberty and adulthood [17, 34, 35], more recent large-scale analyses have paid attention to the characterization of global transcriptomic changes in Kiss1^ARC^ and/or Kiss1^AVPV^ neurons in a variety of conditions. Of note, however, these analyses remain scarce and, in most cases, have concentrated in adulthood. Thus, these studies have highlighted differential patterns of gene expression between these two neuronal populations in adult female mice [36], and illustrated the impact of estrogen administration upon the transcriptome of Kiss1^ARC^ neurons, illuminating pathways with key roles in reproductive control [37]. In addition, the transcriptional profiles of Kiss1^AVPV^ neurons in female mice exposed to fixed doses of estradiol have been described; comparison of transcriptional responses to estrogen in the two Kiss1 populations has identified conserved vs. oppositely-regulated molecular patterns, that strongly suggests differential mechanisms for transcriptional control between Kiss1^ARC^ and Kiss1^AVPV^ neurons [38]. In addition, the active translatome (i.e., whole set of transcripts being actively translated into proteins) of Kiss1^AVPV^ neurons, following estrogen stimulation, has been reported in adult female mice [39]. A similar study has also been conducted in Kiss1 neurons from the amygdala [40].

The above studies, however, did not evaluate global transcriptional changes in Kiss1 neurons during puberty. This aspect has been partially addressed by one recent study, applying spatial transcriptomics to map the dynamic atlas of gene expression of the hypothalamus in female rats across puberty. This allowed the identification of fourteen different cell clusters, including a prominent cluster representative of the ARC, that could be followed from the juvenile (PND-25) to the peripubertal (PND-35) and post-pubertal (PND-45) stages [41]. From a global perspective, distinct expression patterns were found in the hypothalamus during pubertal maturation, suggesting specific developmental programs, including a global decline of γ-aminobutyric acid (GABA) inputs and increase of Glutamatergic inputs [41], in line with classical studies suggesting the lowering of inhibitory signals and the rise of excitatory signals across puberty [9, 10]. Of note, within the ARC cluster, prominent expression of *Kiss1* and other gene markers was found, including also *Tac2* and *Pdyn*; yet, the latter displayed expression patterns that were more scattered across other clusters. In fact, a number of potential ARC gene markers were found, including *Sox14*, *S100g*, *Gck* and *Six6*, whose expression was more specific of the ARC than *Kiss1* itself. Interestingly, comprehensive analysis of gene expression patterns within the ARC cluster allowed identification of four subclusters of neurons, with one harboring the majority of Kiss1^ARC^ neurons [41]. Further analysis of co-expressed markers permitted identification potential markers of Kiss1^ARC^ neurons, with the solute carrier family 18 member A3 (*Slc18a3*) being a solid candidate. Indeed, large Slc18a3^+^ cells were found to highly express *Kiss1*, *Tac2* and *Pdyn*, and were proposed as *bone fide* Kiss1^ARC^ neuronal population, whose number increased across the pubertal maturation [41]. Of note, however, small Slc18a3^+^ cells were also found in the ARC, which apparently correspond to glial cells. While the use of the 10xGenomic Visium platform did not allow for single-cell resolution, these analyses pave the way for a more sophisticated gene mapping of hypothalamic circuits, and particularly Kiss1 neurons at puberty, using a combination of next-gen spatial transcriptomics and RNAseq on specific neuronal populations, at normal and altered conditions.

In addition, recent studies have identified a number of transcription factors that seemingly play crucial roles in Kiss1 neuron fate determination and early development, and hence might contribute to the maturational continuum that ultimately leads to puberty. This is likely the case of Sox14, which is enriched in Kiss1 neurons, and whose ablation results in decreased numbers (but not complete absence) of Kiss1 neurons in the ARC, together with impairment of reproductive indices [42]. In addition, the Nescient Helix-Loop-Helix 2 (Nhlh2), a member of the basic helix-loop-helix family of transcription factors, has been shown to be enriched in Kiss1 neurons and activate *Kiss1* gene expression, while its ablation in Kiss1 cells resulted in delayed puberty, with a preferential impact in males [43]. In addition, it was recently reported that Tbx3, a member of the T-box family of transcriptional regulators, plays a major hierarchical role in maintaining the cellular identity of KNDy neurons, and ablation of Tbx3 in these cells, by using a Tac2-Cre mouse line, resulted in delayed puberty in both male and female mice [44]. Interesting, disruption of neuronal expression of Tbx3 resulted also in alterations of the peptidergic profiles of Proopio-melanocortin (POMC) and Agouti-related peptide (AgRP) neurons, with a detectable impact in terms of body weight gain and metabolic perturbations [45]. This connection is particularly intriguing, given the tight link between body energy status and puberty, as described in detail in Sect. 5 of this review.

### Epigenetic changes and its potential role in Kiss1 neurons and puberty

Besides transcriptional changes in Kiss1 neurons, compelling evidence gathered in the last decade has documented the prominent role of epigenetic regulatory mechanisms in the control of puberty, by operating, at least partially, at the level of Kiss1^ARC^ neurons. Indeed, changes in DNA methylation, histone modifications and microRNAs (miRNAs) have been suggested to participate in the control of different facets of reproduction, including pubertal maturation. Importantly, these epigenetic regulatory mechanisms are not restricted to Kiss1 neurons, and seemingly affect other neuronal cell types, such as GnRH neurons [46], and reproductive tissues [47–49]. Yet, given their physiological relevance, in this section we will focus on epigenetic regulatory pathways converging on Kiss1 neurons.

Seminal findings from the group of Lomniczi and Ojeda paved the way for the characterization of the roles of changes in DNA methylation and histone modifications in the control of puberty [50]. Thus, while the members of the Polycomb Group (PcG) of gene silencers, EED and CBX7, induced a repressive histone configuration that restrains *Kiss1* expression during the juvenile period, increased methylation of the promoters of these genes during puberty led to a decrease in their expression, and hence a rise in *Kiss1* transcription along pubertal maturation, due to a switch in chromatin landscape, from a repressive to a permissive configuration [50]. In parallel, *Kiss1* transcription at puberty is also modulated by mixed-lineage leukemia 1 (MLL1) and MLL3, which belong to the Trithorax group (TrxG) of epigenetic activators of gene expression, which operate in a reciprocal manner to PcG, thus driving pubertal changes in chromatin configuration into a more active form [51]. MML1 activates also NKB gene expression, which may also contribute to its capacity to modulate puberty (see Sect. 4). Other epigenetic players potentially involved in pubertal control are the member of TrxG, CHD7, and the histone demethylating enzyme, KDM6B, although their roles in Kiss1 neurons is yet to be fully defined [52, 53]. In addition, the member of the Zinc Finger (ZNF) family of repressors, GATAD1, displays high levels of hypothalamic expression during the infantile period, that decrease in peripubertal female monkeys, and was capable to suppress *Kiss1* (and NKB) gene transcription, by ultimately decreasing histone activating marks at the promoter level [54]. These findings provide a tenable molecular pathway for the contribution of ZNF genes to the modulation of human pubertal timing, as pointed out by genome-wide association studies [55].

In addition to changes in DNA methylation and histone modifications, pubertal timing is influenced by non-coding RNAs, including prominently miRNAs, operating at the hypothalamus, affecting not only Kiss1 neurons. Initial analyses documented that miRNA pathways, and particularly miR-155 and miR-200, orchestrate a switch in the secretory program of GnRH neurons into an active configuration, that occurs at prepubertal stages and is crucial for proper pubertal initiation [56]. In addition, a recent study has described global changes in hypothalamic content of miRNAs during the pubertal transition, as assayed by small RNAseq in female mice, with four different expression patterns in post-pubertal age, which were associated with different biological pathways, from developmental processes to epigenetic regulation [57]. Regarding regulation of Kiss1 neurons, a recent study from our group documented that genetic ablation of Dicer, as key enzyme for the canonical synthesis of mature miRNAs, selectively in Kiss1 neurons caused a dramatic phenotype of central hypogonadism, in both male and female mice. Yet, onset of puberty was preserved in both sexes after prevention of mature miRNA biogenesis in Kiss1 cells, and only females failed to complete pubertal maturation and attain fertility, whereas males were initially fertile although rapidly progressed into reproductive failure [58]. Molecular analyses in isolated Kiss1 neurons in this model revelated that, preferentially in Kiss1^ARC^ neurons, this reproductive suppression is due to enhanced expression of key *Kiss1* repressors, including Mkrn3, Cbx7 and Eap1, which is bound to reduced expression of *Kiss1* mRNA and kisspeptin content. This further illustrates the multi-layered nature of the regulatory mechanisms controlling puberty, where cascades of repressors of repressors seem to play a crucial role in the proper timing of puberty.

### Changes in the functional electrical properties of Kiss1 neurons during puberty

Post-natal developmental changes in the mammalian brain are often accompanied by synaptic plasticity, such as long term depression or potentiation [59] and/or plasticity of neuronal excitability and the underlying ion channels [60]. Little is known about forms of neuronal plasticity occurring in Kiss1 neurons at puberty. The intrinsic electrical properties of Kiss1 neurons in both the ARC and the AVPV are well characterized in adult male and female mice; for a summary see [61]. In brain slices from adult mice, Kiss1^AVPV^ neurons spontaneously fire action potentials more reliably than Kiss1^ARC^ neurons and at a higher frequency in males compared with females for both populations [61]. Kiss1^ARC^ neurons must sustain action potentials for 2–5 min at a minimum of 10 Hz to increase LH to physiological levels [62], and Kiss1 neurons from adult mice can, when depolarized, fire sustained trains of action potentials at 10–20 Hz (AVPV) and 20–40 Hz (ARC) [61]. However, the firing properties of prepubertal mouse Kiss1 neurons are less well characterized. Kiss1^ARC^ neurons in brain slices from 4–7 day old male and female mice showed sustained action potential firing of around 2 Hz in response to mild depolarization [63]. In recordings from Kiss1^ARC^ neurons in brain slices from female mice aged 18–25 days, 30% fired spontaneously at 2.5 Hz, while all Kiss1^AVPV^ neurons fired spontaneously [64]; a similar incidence of spontaneous firing was observed in Kiss1^ARC^ neurons in brain slices from female mice aged 18–21 days [65]. In these studies of pre-pubertal Kiss1 neurons, the capacity to drive firing to the critical 10 Hz frequency required for LH secretion was not tested.

A recent study reported an increase in the number and duration of action potential firing of Kiss1^ARC^ neurons occurring around puberty in female mice [66]. The increase in firing seen in postpubertal mice was not found if ovariectomy was performed at 3 weeks (with no 17 β-estradiol supplement until 6 weeks), suggesting a window of estrogen-dependent neuronal plasticity of action potential firing. Specific ion channel gene expression changes were also detected across puberty. Notably, between 3 weeks and 6–8 weeks, there was a decrease in Scn2a, encoding Nav1.2 channels, known to influence firing of Kiss1^ARC^ neurons [61], and an increase in Hcn1, encoding hyperpolarization activated cyclic nucleotide-gated channels, which contribute to rebound firing following hyperpolarization in Kiss1 neurons [61, 66]. Whether or not these gene expression changes observed at puberty are also dependent on estrogen is not known and should be explored. Estrogen dependent changes in ion channel gene expression have been reported in Kiss1^ARC^ neurons from adult ovariectomized female mice subjected to estrogen replacement [37, 67], including increased expression of the gene encoding HCN1 channels [67].

The change in firing properties of Kiss1^ARC^ neurons just prior to puberty onset is consistent with a recent finding that pulsatile activity of Kiss1^ARC^ neurons, measured using fiber photometry in female mice, is seen at PND38, i.e., a peripubertal stage. Interestingly, pulsatile activity is bidirectionally regulated by food restriction versus ad libitum food [68]. This is regulated by AgRP neurons in the ARC. This raises another important question to explore: whether synaptic plasticity occurs at puberty. Kiss1 neurons in ARC and AVPV brain slices receive both glutamatergic and GABAergic synaptic inputs [61]. Ionotropic AMPA, kainate and NMDA glutamate receptor agonists evoke calcium signals in male and female Kiss1^ARC^ and Kiss1^AVPV^ neurons [69]. These signals are likely to play important roles in the activation and synchronization of Kiss1^ARC^ neurons [70, 71]. However, their role in the developmental plasticity of Kiss1 neurons at puberty remains unexplored.

As mentioned above, appositions between Kiss1 fibers and GnRH neurons increase during puberty in female mice [30], suggestive of synaptic plasticity. This could reflect a change in the activity of Kiss1 neurons and kisspeptin release, or a change in postsynaptic responsiveness within the GnRH neurons, or both. There is strong evidence that the action potential firing properties of GnRH neurons change during postnatal development: firing frequency measured in brain slices from female mice increased between 1 to 3 weeks of age and then decreased by 4 weeks, decreasing further in adults, and GnRH neurons from 3-week-old mice showed more burst firing compared with adults [72]. The percentage of GnRH neurons that respond to exogenous kisspeptin in brain slice preparations increases from 27% before puberty to 90% after puberty [31]. However, GnRH neurons in brain slices from adult mice were more excitable than in pre-pubertal mice, requiring more depolarizing current to evoke action potentials [73].

## Other key transmitters and pathways for the hypothalamic control of puberty

Recognition of the essential roles of kisspeptins in the control of puberty onset should not eclipse the actual relevance of other transmitters of hypothalamic origin in pubertal regulation; as examples, see [74–76]. In fact, experimental evidence strongly suggest that kisspeptin in not the sole trigger of puberty, but rather a key amplifier for the full expression of the increase of GnRH neurosecretory activity that is mandatory for puberty to proceed normally [77, 78]. Hence, integration and contextualization of kisspeptin signaling within the complex network of central transmitters that control puberty is mandatory for an integral comprehension of the hypothalamic mechanisms of pubertal regulation (Fig. 1).

### KNDy peptides (other than kisspeptins)

As mentioned earlier, Kiss1^ARC^ neurons also express NKB and Dyn, that were proposed to operate within the network of the so-called KNDy neurons as reciprocal regulators of the kisspeptin output onto GnRH neurons, with NKB being stimulatory and Dyn seemingly playing an inhibitory role [79]. This working model, however, has been partially revised recently [19]. In one hand, in line with the view that at least a fraction of Kiss1^ARC^ neurons are glutamatergic, glutamate signaling via AMPA receptors has been proposed as major synchronizing factor for the network of KNDy neurons, whereas NKB would act as potentiator of glutamate-mediated synchronization [70]. In turn, the role of Dyn signaling as a “brake” for kisspeptin release has been disputed by recent work in mice, showing that selective ablation of K-opioid receptors (KOR) in Kiss1 cells failed to disrupt LH pulsatility, as surrogate marker of the endogenous secretory profile of kisspeptins [80]. Alternatively, Dyn has been proposed as a potential gating factor, setting the probability of synchronization events within the KNDy network [70]. Of note, however, differences may exist in the relevance of the different KNDy factors across species, as illustrated by the clear inhibitory effects of Dyn in sheep [19].

While the above studies were conducted mainly in adulthood, the roles of NKB and Dyn in the control of puberty have been also addressed by a combination of genetic and pharmacological experiments. The hypothalamic expression of *Tac2* and the gene encoding NKB receptor, *Tacr3*, increases during pre-pubertal maturation in female rats and mice [35, 81], while administration of the NKB receptor agonist, senktide, enhanced LH secretion and advanced puberty onset [35, 82]. In turn, blockade of NKB signaling deferred puberty in female rats [35, 83]. In line with these pharmacological findings, genetic inactivation of NKB resulted in delayed puberty in female, but not male mice [84], suggesting a more predominant role of NKB in the control of female puberty. In line with these findings, stimulatory LH responses to senktide were persistently detected across postnatal development only in female rats, while in males no LH responses were observed from puberty onwards [85]. On the other hand, Dyn might play an inhibitory role in pubertal control, as suggested by the fact that administration of a KOR antagonist caused accelerated puberty onset and increased pulsatile LH secretion in female rats [82]. However, mice with conditional ablation of KOR in Kiss1 cells did not display detectable pubertal alterations [80], arguing against an indispensable role of Dyn signaling in Kiss1 neurons in the precise control of puberty.

### Other tachykinins and neuropeptides

NKB belongs to the family of tachykinins (TAC), which includes also Substance P (SP) and Neurokinin A (NKA), that are both encoded by the *Tac1* gene and operate via the TAC receptors, NK1R and NK2R [86]. Compelling evidence has documented that SP and NKA cooperate with NKB in the control of Kiss1^ARC^ neurons, albeit with potential redundant actions and via different neuronal pathways [87]. In terms of pubertal regulation, it has been reported that hypothalamic *Tac1* expression reaches maximal levels prior puberty, while central agonism of NK1R induced LH secretion and accelerated puberty in immature female mice [88]. In turn, genetic inactivation of *Tac1* delayed puberty onset in male and female mice [88, 89]; yet, part of this phenotype may stem from ablation of NKA production, which is also encoded by Tac1. Although part of the stimulatory effect of SP is likely conducted at the level of a subset of Kiss1^ARC^ neurons, it has been reported that NK1R and the kisspeptin receptor can heterodimerize, making tenable that SP and kisspeptin also interact on GnRH neurons [89]. In addition, pharmacological experiments have suggested a role of NKA in pubertal modulation, since chronic central activation of NK2R in immature female mice led to advancement of the age of puberty onset [90]. However, studies in a genetic model of congenital ablation of NK2R, encoded by *Tacr2*, did not alter pubertal timing in male or female mice [91]. While we cannot exclude the possibility that NKA signaling is totally dispensable for pubertal regulation, these findings might be compatible also with some degree of redundancy on their pubertal roles of among different TAC, as supported also by electrophysiological analyses of Kiss1^ARC^ responses in adult mice [86].

While TAC carry out predominant stimulatory effects, central inhibitory transmitters have been claimed to play also a relevant role in the control of puberty. Besides the functions of GABA, as the main inhibitory neurotransmitter in the brain [92], and Dyn (reviewed in Sect. 4.1.), the gonadotropin-inhibitory hormone (GnIH), initially identified in birds and later detected as the mammalian homolog peptides, RF-amide related peptides (RFRP), has been proposed to operate in the central control of the HPX axis [93], as signal to counter-balance the potent stimulatory effects of factors, such as kisspeptins. While the actual physiological roles of RFRP-3, as the main representative of GnIH in mammals, in the central regulation of GnRH secretion is still under debate, some studies have addressed its role in the control of puberty. Thus, genetic ablation of the main RFRP-3 receptor, NPFF1R, did not affect pubertal timing in mice; yet, prepubertal males displayed elevated LH levels that returned to normal levels after puberty [94]. On the other hand, chronic activation of RFRP-producing neurons using activator Designer Receptor Exclusively Activated by Designer Drugs (DREADDs) delayed puberty onset in male (but not female) mice and perturbed reproductive cycle progression in female mice [95]. These data support a modulatory role of RFRP signaling in the control of puberty, although its physiological relevance is yet to be clarified.

Other neuropeptide systems have been disclosed in recent years as putative components of the hypothalamic circuits controlling puberty. A clear example is the pathway originating from the ventral premammillary nucleus (PMv), formed by neurons expressing the pituitary adenylate cyclase-activating polypeptide (PACAP). These neurons project to Kiss1^ARC^ and Kiss1^AVPV^ neurons, and ablation of this circuit delayed the onset of puberty in female mice, suggesting that PACAP-expressing neurons from the PMv modulate pubertal timing via regulation of Kiss1 neurons [96]. Given the role of PMv in transmitting the reproductive effects of leptin [97], this pathway may contribute to the metabolic control of puberty, especially because this population of PACAP^PMV^ neurons express leptin receptors; for further details, see Sect. 5 of this review. Similarly, central irisin signaling has been recently shown to participate in the regulation of puberty. Irisin is a factor secreted from the muscle, whose levels are reported to increase during pubertal transition. Pharmacological blockade of irisin actions or ablation of irisin receptors in forebrain neurons resulted in pubertal delay; a phenotype observed also after global knock-out of the irisin-encoding gene, *Fndc5* [98]. Intriguingly, another muscle-born signal, myostatin, has been recently identified as putative regulator of FSH secretion [99]; however, its eventual role in pubertal control is yet to be defined.

### Nitric oxide

Nitric oxide (NO) is a gaseous transmitter whose role in the control of GnRH secretion was proposed more than three decades ago [100]. Analysis of the reproductive roles of NO has gained momentum recently, with the recognition that heterozygous loss-of-function mutations in the gene encoding neuronal NO synthase, *NOS1*, are associated to hypogonadotropic hypogonadism in humans [101], while mice deficient for *Nos1* are known to display reproductive defects [102] and suffered delayed puberty onset in both sexes [101]. Further characterization of the roles of NO synthesis revealed that Nos1 activity is essential to shape minipuberty in female mice during the infantile period, a key process for later pubertal maturation [101]; male minipuberty in mice appears to be modulated also by Nos1 activity in the preoptic region [103]. A tenable pathway for the central reproductive actions of NO involves the interplay between Kiss1-, NO- and GnRH-producing cells, the so-called “KiNG” network, in which kisspeptin and NO reciprocally operate to modulate the output of GnRH neurosecretion, with NO playing a predominant inhibitory role [104], which has been also substantiated in previous pharmacological studies [105].

### Makorin Ring Finger Protein 3 (MKRN3)

The crucial role of MKRN3 in pubertal control was initially exposed in 2013, when human genetic studies documented that heterozygous mutations of this gene were associated with central precocious puberty (CPP) [106]. This finding was later confirmed and extended by numerous studies that have documented that mutations of MKRN3 are the most frequent cause of monogenic CPP [107]. This clinical phenotype strongly suggested that MKRN3 plays a repressive function in the control of puberty, which is also supported by the dramatic drop in Mkrn3 mRNA expression in the hypothalamus of mice and rats preceding puberty [106, 108]. Accordingly, overexpression of Mkrn3 in the mediobasal hypothalamus resulted in delayed puberty in female mice, along with decreased content of kisspeptin and NKB [109]. Despite this strong evidence, the ultimate mechanisms for the pubertal regulatory actions of MKRN3 are yet to be fully clarified. It has been proposed that Mkrn3 operates in Kiss1^ARC^ neurons in the mouse and has the capacity to repress human *KISS1* and *TAC3* (encoding NKB) promoters [110]. Besides this transcriptional repression, Mkrn3 is endowed with ubiquitinase activity, which may play a role also in degradation of puberty-related proteins. Protein targets of hypothalamic Mkrn3 include NKB and insulin-like growth factor 2 mRNA-binding protein 1 (IGF2BP1) [111]; the mechanistic pathway whereby the later interaction modulates puberty is yet to be disclosed. As an additional mechanism, studies in human hypothalamic neurons derived from iPSC suggested that MKRN3 may regulate puberty by affecting gene programs affecting hypothalamic plasticity and development [111]. Interestingly, the prepubertal drop of Mkrn3 in rat female hypothalamus is controlled by miR-30, whose levels increased before puberty; thus, prevention of the repressive effects of miR-30 on Mkrn3 during the juvenile period delayed puberty onset [108]. These reciprocal changes in the expression levels of Mkrn3 and miR-30 preceding puberty have been also detected from their circulating levels in prepubertal boys [112, 113]. The physiological relevance of such peripheral changes of the miR-30/Mkrn3 system in human puberty remains unknown.

### Non-neuronal cells and the control of puberty: key roles of astrocytes and tanycytes

Besides multiple neuronal inputs (see previous sections), compelling evidence has documented a prominent role of non-neuronal, glial cells in the precise control of puberty. In this context, initial data supporting a role of astrocytes in the control of pubertal timing was provided in early 1990’s, as putative mechanism for acceleration of puberty following brain injury [114]. Since then multiple, studies have conclusively demonstrated a major role of astrocytes in pubertal control, as extensively reviewed elsewhere [115]. As most prominent recent findings, it has been recently shown in mice that GnRH neurons recruit astrocytes during infantile period via a prostaglandin-DP1 signaling pathway, whose disruption impairs minipuberty and delays sexual maturation [116]. In addition, it have been demonstrated that pubertal acceleration, due to early exposure to a high-fat diet (see Sect. 5), is accompanied by an increase in astrocyte–GnRH neuron interactions [117], and that astrocytes are capable to activate GnRH activity and LH release in mice, independent of ARC Kiss1 neurons [118]. Intriguingly, tanycytes, specialized ependymoglial cells lining the third ventricle, have been recently shown to play also a very active role in the dynamic control of reproduction, and its gating by metabolic cues. Thus, estrogen receptor signaling in tanycytes, via estrogen receptor-α, plays a role linking estrogenic feedback to orexigenic NPY neurons and is required for normal LH pulsatility, estrous cyclicity and the anorexigenic effects of estrogen [119]. In addition, depletion of glial fibrillary acidic protein (GFAP)-positive tanycytes caused a dramatic suppression of GnRH neurons and LH secretion, leading to hypogonadotropic hypogonadism in male mice [120]. Despite this compelling evidence, the actual role of tanycytes in the dynamic control of puberty, and their interplay with its hormonal and neuronal regulators, is yet to be defined and warrants further investigation.

## Metabolic control of puberty – major players and hypothalamic pathways

**Fig. 3:**
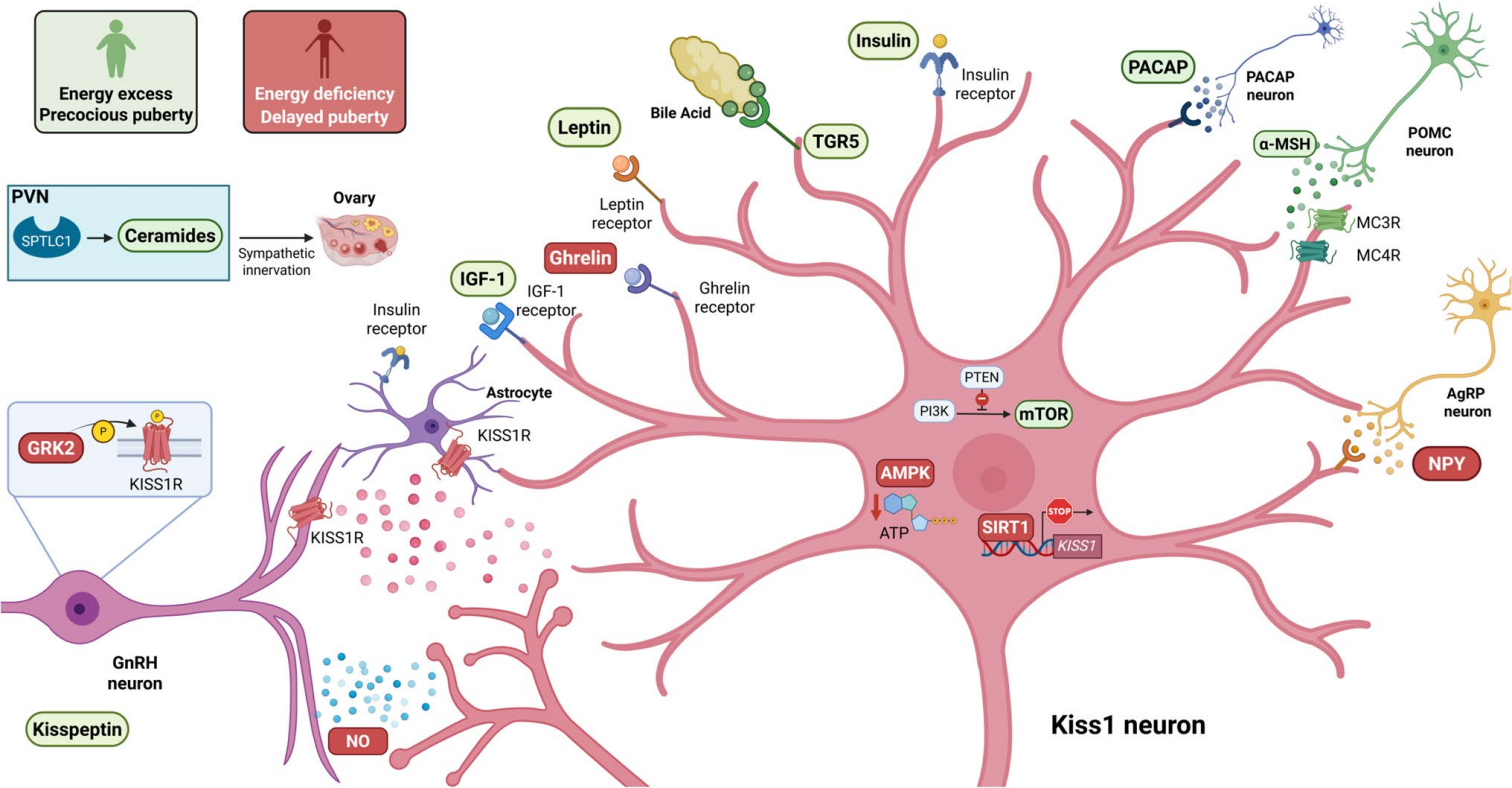
Overview of the neuroendocrine mechanisms for the metabolic control of puberty. Schematic representation of the variety of signals and regulatory mechanisms, converging on Kiss1 neurons, that have been reported to contribute to the metabolic control of puberty. Signals that are predominant in conditions of energy insufficiency are labelled in red, while factors that predominate in conditions of energy excess are marked in green. The contribution of intracellular mechanisms involving cell energy sensors, as well the role of non-synaptic inputs, including the recently described Kiss1r-signaling pathway in astrocytes, are depicted. Other pathways, such as the one involving de novo ceramide synthesis and sympathetic innervation of the ovary, are also shown. For further details, see Sect. 5. Created with BioRender®

Metabolic signals and nutritional status are among the most prominent modifiers of puberty. Thus, extreme metabolic conditions and severe changes in body weight, ranging from undernutrition to obesity, are bound to changes in pubertal timing [97, 121]. This tight connection between puberty and body energy reserves was assumed on the basis of intuitive knowledge for centuries, but was scientifically formulated only in 1960’s-70’s, based first on the seminal work of Kennedy and Mitra using animal models, that was followed by the proposal of the critical fat mass hypothesis in humans by Frisch and co-workers [122]. To a large extent, the interplay between metabolic status and pubertal maturation takes place at the hypothalamus, where peripheral and central metabolic signals interact, via intricate pathways, with the hypothalamic circuits governing puberty onset [121]. In recent decades, our knowledge of these neuro-endocrine pathways has enlarged substantially, including the characterization of the pubertal roles of peripheral hormones with key metabolic functions, including leptin, ghrelin and insulin, and the recognition of Kiss1 and other neuronal circuits, as key components for the transmission of metabolic information to central puberty-controlling systems [3]. While detailed recapitulation of recent progress in this area is beyond the scope of this review and can be found elsewhere [2, 121], we will briefly highlight in this section some illustrative recent advancements in our knowledge of the central mechanisms whereby nutritional status and metabolic signals can modulate pubertal timing (Fig. 3).

### Metabolic hormones and the control of puberty: mechanisms of action

There is ample consensus that key metabolic hormones can influence puberty onset, with opposite roles between anorexigenic factors, that operate as signals of energy abundance and drive a positive or permissive role on pubertal progression, such as leptin and insulin, and orexigenic factors, such as ghrelin, that signal conditions of energy deficit and exerts a negative influence on puberty onset [121]. The mechanisms whereby these hormones convey their actions onto GnRH neurons remains debatable, but solid experimental evidence points out that this is conducted indirectly by modulation of afferents to GnRH neurons. In this context, recognition of Kiss1 neurons, particularly Kiss1^ARC^, as major hub for the tonic control of the HPG axis led to the hypothesis that these operate also as a major hub for transmitting the actions of these peripheral hormones to GnRH neurons. This contention, however, has been disputed by studies using functional genomics to congenitally ablate key receptors, such as leptin (LepR) or insulin (IR) receptors from Kiss1 cells, which showed largely conserved pubertal progression. Considering that conditions of metabolic impairment linked to reduced leptin or insulin levels are bound to decreased *Kiss1* expression, these findings suggest an indirect mode of action, via upstream afferents to Kiss1 neurons, which may include leptin-sensitive NO-producing neurons in the preoptic area [123], and well as PACAP- and dopamine transporter (DAT)-expressing neuronal projections from the PMv [96, 124]. Recently, these findings have been complemented by studies where the receptor for IGF-1 (IGFR) was congenitally ablated from Kiss1 cells. Mice of both sexes lacking IGF-1 signaling in Kiss1 neurons displayed severely delayed puberty, together with clear metabolic and growth alterations [125], supporting that, in contrast to leptin and insulin, direct actions of IGF-1 on Kiss1 neurons are indispensable for proper pubertal progression. In addition, conditional elimination of insulin receptors from astrocytes also caused delayed puberty [126], together with severe impairment of reproductive function in both sexes, thus indicating that insulin sensing in these glial cells plays a relevant role in pubertal control. Altogether, these and previous findings indicate that metabolic hormone signaling converge at different neuronal (including Kiss1) and non-neuronal pathways controlling GnRH neurons to precisely modulate puberty and adjust its progression to metabolic status.

### Central metabolic pathways and puberty: Prominent roles of POMC and AgRP neurons

While there is no doubt that Kiss1^ARC^ neurons participate in the transmission of metabolic information to GnRH neurons, whether metabolic signals operate directly on this neuronal population or upstream afferents is still matter of debate. Recent developments have solidly documented that, regardless of the possibility of direct actions on Kiss1 neurons, other metabolically-sensitive neurons in the ARC, notably including POMC^ARC^ and AgRP^ARC^ neurons, do play a relevant role in the metabolic control of Kiss1^ARC^ neurons and, thereby, pubertal maturation. Thus, very elegant work published recently by Goto and colleagues has illustrated not only that the functional activity of Kiss1^ARC^ neurons, as detected by synchronization episodes in fiber photometry registries, is suppressed by conditions of early undernutrition that are bound to delayed puberty, but also that AgRP^ARC^ neurons, which become activated in conditions of hunger, have a very relevant role in this phenomenon [68]. These observations are in line with previous reports in adult mice showing that AgRP^ARC^ neurons play a crucial role in functional suppression of Kiss1^ARC^ neurons in conditions of energy deficit and starvation [127]. Similarly, Neuropeptide Y (NPY), which is also expressed in AgRP^ARC^ neurons and is induced by fasting, has been shown to suppress Kiss1^ARC^ neurons in male and female mice [128].

In addition, POMC^ARC^ neurons, which are activated in conditions of energy surplus, have been proposed also to play a major role in the metabolic control of puberty. POMC^ARC^ neurons produce melanocortins, such as α-MSH, which has been shown to modulate Kiss1^ARC^ neurons to drive a stimulatory action on puberty onset and LH secretion in immature female rodents [129]. This pathway seems to be essential for transmitting the permissive effects of leptin on puberty onset. The relevance of melanocortin signaling in Kiss1^ARC^ neurons has been recently substantiated by functional genomic studies, that showed that ablation of melanocortin 4 receptors (MC4R) from Kiss1 neurons induced reproductive impairments in females without causing overweight, whereas reinsertion of MC4R in Kiss1 neurons from global MC4R KO mice rescued ovarian cyclicity [130]. While these data collectively point out a major role of MC4R signaling in the integration of metabolic status, puberty and reproductive function, it must be noted that solid genetic data in humans and experimental observations in rodents, including from MC3R null mice, have documented a conserved role of MC3R signaling, involving also Kiss1 neurons, in the joint control of the nutritional status and pubertal timing [131].

As a final comment in this section, kisspeptins not only interplay with other neuronal circuits for the putative modulation of puberty by metabolic signals, but also engage with non-neuronal afferents (see also Sect. 4.5). In this context, in a recent study, Torres and co-workers have documented a novel kisspeptin signaling pathway, mediated by the canonical receptor, Kiss1r, in astrocytes, that participates in the fine tuning of different aspects of the HPG axis [132]. Notably, ablation of Kiss1r in Gfap-expressing astrocytes partially prevented the impact of early-onset obesity on pubertal acceleration in female mice. These findings strongly suggest that kisspeptin signaling may not only contribute to the metabolic modulation of puberty by direct actions on GnRH neurons, but also via indirect actions on astrocytes, as key non-neuronal regulators of GnRH neurosecretion and puberty onset [132].

### Central energy sensors and pubertal control: Roles of mTOR, AMPK and

The data summarized in previous sections delineates putative neuroendocrine pathways that convey the modulatory actions of key metabolic signals onto GnRH neurons, as the final output circuit for pubertal activation. This is illustrated by, but not restricted to, the pathway involving leptin → POMC^ARC^ → Kiss1^ARC^ → GnRH neurons, as described above. Notwithstanding, compelling evidence accumulated over the last decade has documented that, in addition to these neuroendocrine signals, a variety of cellular energy/metabolic sensors and multiple mediators seemingly cooperate in the physiological control of puberty and its alterations in adverse metabolic conditions.

Experimental data obtained mainly in rodent models have demonstrated that two essential cell energy sensors, namely the mammalian target of rapamycin (mTOR) and AMP-activated protein kinase (AMPK), operating in Kiss1^ARC^ neurons or their afferents, play a key role in transducing metabolic information to puberty-regulating circuits within the hypothalamus. Of note, mTOR and AMPK in key hypothalamic nuclei, such as the ARC and the ventromedial hypothalamus, have been shown conduct relevant roles, in a ying-yang fashion, in the central control of body energy balance, by controlling food intake and thermogenesis [133, 134]. In a similar manner, mTOR and AMPK would reciprocally operate to adjust pubertal timing to body energy status. Thus, using preclinical rodent models, we have shown that preserved brain mTOR signaling, which is activated in conditions of energy excess and high leptin levels, is mandatory for normal pubertal progression and absolutely required for the transmission of the permissive effects of leptin on puberty onset in female rats [135]. Conversely, AMPK activity, which is enhanced in situations of energy deficit, cooperates in the suppression of female pubertal maturation observed in conditions of undernutrition, via actions on Kiss1 neurons [136].

In addition, the metabolic sensor, SIRT1, the best known member of the family of sirtuins, has been proposed also as putative link between metabolic status and puberty, acting on hypothalamic Kiss1^ARC^ neurons. Like AMPK, SIRT1 is activated in situations of energy deficit [137], and is expressed in the ARC, with elevated expression after food deprivation [138]. The hypothalamic content of SIRT1 declines during pubertal maturation and central pharmacological activation of SIRT1 not only suppresses *Kiss1* expression in the hypothalamus but delays also puberty onset in female rats; an effect mimicked by virogenetic overexpression of SIRT1 in the ARC [139]. Notably, immunohistochemical content of SIRT1 increased in Kiss1^ARC^ neurons in conditions of undernutrition, associated with pubertal delay. Altogether, these findings strongly suggest that SIRT1 may operate as a repressor of *Kiss1*, as mechanism for pubertal suppression in conditions of negative energy balance. Considering that SIRT1 is a NAD^+^-dependent class III deacetylase, with capacity to induce histone modifications [137], SIRT1 can be considered a key component of the epigenetic machinery that connect pubertal control and nutritional status [139]. In fact, chromatin-immunoprecipitation (ChIP) assays have documented the repressive action of SIRT1 at the Kiss1 promoter, so that eviction of SIRT1 from this promoter allows a repressive-to-permissive switch in the chromatin landscape during pubertal progression. This repressive configuration is protracted in conditions of undernutrition due to retention of SIRT1 at the *Kiss1* promoter. Conversely, hypothalamic SIRT1 content and the levels of SIRT1 in Kiss1^ARC^ neurons are increased in conditions of obesity linked to accelerated puberty in female rats; i.e., opposite to what is observed in situations of energy deficit. Early-onset obesity accelerated the eviction of SIRT1 from the Kiss1 promoter, as putative mechanisms for heightened Kiss1 expression and advanced puberty in situations of overweight [139]. Besides these actions in Kiss1^ARC^ neurons at puberty, SIRT1 operating on Kiss1^AVPV^ neurons seems to play a role in gating the pre-ovulatory surge of gonadotropins and its suppression in conditions of energy deficit [140]. Of note, over-expression of a deacetylase-deficient SIRT1 mutant in astrocytes has been recently shown to suppress *Kiss1* expression and impaired several reproductive indices in female mice, suggesting that non-neuronal SIRT1 may also contribute to the regulation of *Kiss1* and reproductive function in an indirect manner [141]. In addition, we have recently demonstrated that, besides Kiss1 neurons and glial cells, GnRH neurons themselves are also equipped with energy sensing mechanisms, involving AMPK and the G-protein-coupled receptor kinase, GRK2, that would contribute also to pubertal delay in conditions of energy deficit [142, 143].

### Central lipid sensing and the metabolic control of puberty

In addition to the prominent roles of the cellular energy sensors described in the previous section, mounting evidence strongly suggests that relevant lipid mediators and lipid sensing mechanisms, operating in the hypothalamus, play also relevant roles in the metabolic control of puberty [144]. In this context, it has been recently proposed that the central systems involved in metabolic and body weight homeostasis are not only sensitive to global changes in body energy status but can sense and react to changes in specific lipid signals, thus defining the concept of hypothalamic lipid sensing [144]. Yet, the putative role of such lipid sensing mechanisms in pubertal control has begun to be disclosed only recently. As an illustrative example, our group has documented that brain de novo ceramide synthesis participates in the control of puberty and particularly, in pubertal acceleration due to early obesity [145]. Thus, postnatal overfeeding caused an increase in the hypothalamic content of ceramides and enhancement of endogenous brain ceramide levels induced pubertal acceleration in lean female rats, while central inhibition of ceramide synthesis delayed puberty and blocked the effects of kisspeptins [145]. This action is seemingly conveyed via a GnRH-independent pathway, involving Kiss1 neuronal projections to the paraventricular nucleus (PVN), where the key enzyme for ceramide synthesis, serine palmitoyltransferase long chain base subunit 1 (SPTLC1), is induced by early overweight and its PVN-specific suppression prevents pubertal acceleration due to obesity [145]. These ceramide-synthesizing neurons in the PVN are seemingly the origin of a novel pathway involving direct sympathetic innervation of the ovary, as the ultimate mechanism for advanced pubertal maturation in conditions of obesity selectively in females.

More recent studies have documented large-scale lipidomic changes in the hypothalamus of peri-pubertal female rats and have disclosed the putative contribution of other lipid sensing mechanisms, including receptors for free fatty-acids (FFAR), peroxisome proliferator-activated receptors (PPAR) and the bile-acid (BA) receptor, TGR5, in the control of puberty [146]. Interestingly, while lipidomic changes in the hypothalamus of maturing rats were predominantly driven by age, a discrete subset of lipid species, including several fatty-acyls, BA derivatives and several glycerol(phospho)lipids, were mainly affected by the obesogenic diet or the interaction between age and diet. In addition, central PPAR signaling and TGR5 modulated pubertal timing, with modest effects also of some FFAR components, such as GPR84, as evidenced by pharmacological studies. Notably, the pubertal actions of the components of the lipid sensing machinery occurred in a variable manner depending on the maturational and nutritional status. Thus, while central blockade of PPAR-γ/α was capable to delay puberty preferentially in lean female rats, central stimulation of TGR5 signaling partially prevented obesity-induced advanced puberty, but was largely ineffective in conditions of normal nutrition [146]. The putative role of changes in circulating BA levels and hypothalamic TGR5 signaling in the central regulation of female puberty has been suggested also by a very recent study combining clinical and preclinical data, which showed that TRG5 activation can induce GnRH secretion in a kisspeptin-dependent manner, while overexpression of TGR5 in the ARC advanced the onset of female puberty [147]. Of note, hypothalamic BA signaling via TGR5 has been recently recognized as a key component for metabolic homeostasis [148], thus suggesting that this pathway is likely to also play a relevant role in the central mechanisms for the joint control of puberty and metabolism.

## Hypothalamic control of puberty and implications for pubertal alterations

The hypothalamic mechanisms summarized in previous sections can explain not only the basis for the physiological control of puberty, but may also shed light onto the pathophysiological mechanisms of pubertal alterations. This is considered relevant for two main reasons. First, consistent changes in the age of pubertal initiation have been recently reported across numerous studies, with a trend for acceleration of the mean age of puberty onset affecting preferentially girls [149], but possibly also boys [150]. Second, even within the normal range, variations in the timing of puberty have been linked to adverse health outcomes later in life, with advanced or delayed pubertal timing being associated to higher risk of a wide variety of conditions, from cardiometabolic diseases to reproductive disorders and some types of cancer, in women and men [151]. Since the mechanisms for the above population changes in pubertal age and their consequences remain ill defined, deepening our knowledge of the circuits responsible for the regulation of puberty seems mandatory as basis for better understanding pubertal disorders and their management. While much progress has been made on the characterization of the genetic basis of pubertal alterations [2, 107], in this section we will revise some paradigmatic examples on how metabolic conditions and non-genetic modifiers can interplay with hypothalamic circuits for the pathophysiological regulation of puberty.

As mentioned earlier in this review, a major component for the variations in pubertal age comes from nutritional factors, with conditions ranging from undernutrition to obesity causing delayed or accelerated puberty, respectively. While the hypothalamic mechanisms for such effects are revised in detail in Sect. 5, it is worth mentioning that specific nutritional components as well as endogenous players, such as the microbiota, have recently been shown to contribute to pubertal modulation. As an example of the former, recent but as yet fragmentary and somewhat conflictive evidence has been presented for the impact of artificial sweeteners on pubertal maturation. Thus, a very recent study investigated the potential impact of aspartame consumption on female puberty, based on clinical and preclinical data. These results showed that chronic aspartame consumption delayed puberty in female rats, while in girls, aspartame intake was associated with a lower risk of precocious puberty [152]. While part of this action might involve peripheral deregulation of mitochondrial function at the ovarian level, aspartame may suppress central expression of GnRH, Kiss1 and Kiss1r, while increasing the expression of the inhibitory signal, RFRP, at the hypothalamus, as putative mechanism for pubertal delay [153]. These data, however, should be taken with caution until more comprehensive analyses of the relevance of doses tested and mechanisms involved are reported.

Solid data indicate that physical activity, within normal ranges, and other lifestyle habits can have an impact per se on the timing of puberty in humans. Thus, results from the UK Millennium Cohort Study point out that elevated total daily physical activity, regardless of its intensity, was linked to a reduction in the risk of early puberty, independently of changes in body mass, especially in girls [154]. Conversely, a regional study conducted in a school-based cohort of 3650 children documented that increased sedentary behaviors elevated the risk of accelerated puberty in girls, whereas time of exposure to screen light per se did not have a discernible impact on pubertal timing [155]. It must be noted, however, that the mechanisms for pubertal modifications due to physical activity (or lack of) are yet to be fully clarified and can intersect with those of energy balance at central levels, since, as revised in previous sections, over- and underweight during the infantile/prepubertal period can have a profound influence on the hypothalamic pathways controlling the tempo of puberty.

It is well known that different metabolic perturbations can have a substantial impact on pubertal maturation, due to the alterations of some of the neurohormonal circuits reviewed in previous sections. As paradigmatic example, uncontrolled type 1 diabetes (T1D) is linked to higher risk of delayed puberty and menarche, together with oligomenorrhoea and lower fecundity later in life [156]. A tenable mechanism for such a connection is the suppression of the Kiss1 system in conditions of negative energy balance, as suggested by preclinical studies [156]; yet, the contribution of other neuroendocrine pathways, described in Sect. 5, cannot be discarded and merits future investigation. Conversely, earlier age at menarche is associated to higher risk of type 2 diabetes (T2D) or impaired glucose tolerance, via mechanisms that are yet to be fully defined [157], but seem to be independent of adiposity [158].

Regarding the microbiota, different studies have been published recently connecting changes in pubertal age and modifications in gut microbiome, both in humans and experimental animals [159, 160]. Of note, the mechanisms whereby the microbiota can influence puberty are likely diverse and may include both direct actions of microbiota-derived signals on the central circuits for pubertal control as well as indirect effects via modification of metabolic status. On the latter, it has been well documented that the diet is a major determinant of microbiota composition and perturbations in the intestinal microbiome can predispose to development of obesity, which, as explained earlier, can profoundly affect pubertal timing on its own. Nonetheless, solid evidence suggests more direct mechanisms of action as well, including changes in BA levels and central TGR5 signaling [146, 147], and modifications in the levels of short chain FA, such as butyrate and acetate, derived from the gut microbiome, which are thought to operate, at least partially, via modulation of the kisspeptin-GnRH system [160]. Of note, a very recent study has documented elevated levels of the bile acid, muricholic acid (MCA), in girls with central precocious puberty, and peak serum concentrations of MCA during pubertal maturation in healthy children; a tenable mechanism for the potential effects of MCA on puberty being its capacity to modulate GnRH secretion via TGR5 [161]. In any event, the pathophysiological relevance of this gut-brain connection in the control of normal and perturbed puberty is yet to be fully defined.

In addition, ample literature has documented the impact of environmental compounds with endocrine-disrupting activity (EDC) on pubertal maturation, by acting, at least partially, on key hypothalamic circuits. While detailed recapitulation of the impact and central mechanisms of action of EDC on pubertal regulation is beyond the scope of this review and can be found elsewhere [162], it is worth mentioning that prominent EDCs, such as bisphenol A (BPA), have been shown to modulate key components of the puberty-controlling pubertal pathways, as Kiss1 neurons [163, 164]. The effects of BPA can be detected even at low, environmentally-relevant doses and appear to be non-monotonic, with opposite effects beings observed for low vs. high doses [165]. Moreover, BPA can influence pubertal maturation of both Kiss1^ARC^ and Kiss1^AVPV^ neuronal populations in opposite manners [163], and can perturb hypothalamic gliogenesis during the infantile period, as a key phenomenon in pubertal control [116], whereas relevant mixtures of EDC has been shown to have a profound transgenerational impact on pubertal timing, by epigenetic mechanisms affecting Kiss1 expression [166]. Finally, the possibility that kisspeptin levels may serve as a putative biomarker for exposure to mixtures of EDC has been recently proposed based on a pilot study in adolescent boys [167]. Altogether, these data raise a call of concern for the potential durable impact of early developmental exposures to EDCs on pubertal maturation, via the disruption of key hypothalamic circuits.

## Conclusions and future directions

Puberty is a fascinating developmental event, sensitive to genetic, endogenous and environmental factors. Although the timing of puberty is to a large extent genetically determined and shows a notable stability across populations [2], the precise definition of the age of pubertal maturation results from the dynamic interplay between core components of the hypothalamic pathways governing the reproductive axis, together with endogenous hormones, peripheral signals and external cues. Our knowledge of the hypothalamic circuits that carry out this fundamental function has expanded dramatically in recent years, as epitomized by the characterization of the roles of Kiss1 neurons in the control of puberty. However, our understanding of how different neuronal subpopulations (Kiss1 and other neurons) interact for the precise control of puberty remains incomplete. In this sense, the growing recognition of the importance of neuronal heterogeneity within supposedly uniform populations, such as POMC or Kiss1 neurons [7, 168], will likely guide future research lines directed towards the elucidation of the pubertal relevance of such heterogeneity, and to what extent this might be influenced by early developmental conditions, including changes in nutrition and metabolic state. These analyses may help to explain not only the subtleties of pubertal regulation and interindividual variations within the range of normality, but also the basis of some pubertal alterations in adverse conditions.

Despite the astonishing progress in the molecular profiling of different hypothalamic populations in adulthood [169], the transcriptomic patterns of key puberty-controlling cells during pubertal maturation remain largely unknown and, opposite to recent advancements in the adult mouse and human hypothalamus [170, 171], the transcriptomic landscape of the maturing hypothalamus has begun to be disclosed only recently [41]. In this context, implementation of next-generation spatial transcriptomic analyses will help to shed light into the complex interplay between different neuronal and non-neuronal pathways within the hypothalamus particularly during the window of pubertal maturation, and how these might be affected by early modifiers of puberty, from nutritional challenges to EDC exposures.

Substantial progress in the functional characterization of the hypothalamic circuits governing key neuroendocrine functions, including fertility, have been grounded on key methodological advancements that are optimized mostly for application in adult animals. This is illustrated by the use of virogenetic tools to manipulate specific neurons or circuits, or the possibility to register neuronal activity using fiber photometry [172, 173]. In the same vein, novel techniques for in vivo gene editing based on the CRISPR-Cas technology have been applied recently to Kiss1 neurons in adult rodents [174, 175]. However, successful implementation of similar approaches in neonatal or immature experimental animals is not trivial and require extensive optimization, including appropriate stereotaxic administration and assessment of vector expression dynamics in ages preceding puberty. While advancements have taken place recently in this front [68], further development of these tools is needed to precisely interrogate the role of the various components of the hypothalamic circuits controlling puberty in a timely manner.

Finally, while human and experimental studies have substantially advanced our knowledge of the factors and mechanisms involved in pubertal control, more comprehensive analyses, aiming to integrate clinical findings with relevant preclinical data, are needed to set the basis for a more rational and personalized approach to manage pubertal alterations. For instance, mechanistic interrogation of gene variants with proposed roles in pubertal regulation based on genetic studies, using not only rodent but also non-rodent (e.g., Zebrafish or Drosophila) models when relevant, can provide information of high translational value. Similarly, mechanistic analyses directed to elucidate the molecular basis for the long-term health risks bound to alterations in pubertal timing are needed, and specific studies directed towards the characterization of the neuroendocrine mechanisms for obesity-induced pubertal perturbations should be conducted to define the putative substrate for the current trends of changes in pubertal age across different populations, in the face of the escalating prevalence of child obesity worldwide. These and related open questions set the scene for a fertile area of research, that holds the promise for exciting developments in the near future.

## Data Availability

No datasets were generated or analysed during the current study.
